# Acute coronary syndrome complicating infective endocarditis: A case report with an etiological review

**DOI:** 10.1016/j.amsu.2022.104737

**Published:** 2022-09-22

**Authors:** Soumia boulouiz, Amine Bouchlarhem, Saidia Amaqdouf, El ouafi Noha, Zakaria Bazid

**Affiliations:** a Faculty of Medicine and Pharmacy, Mohammed I^st^ University, Oujda, Morocco; b Department of Cardiology, Mohammed VI University, Hospital Mohammed I University, Oujda, Morocco; c Mohammed First University, Faculty of Medecine and Pharmacy, Oujda, Morocco

**Keywords:** Infective endocarditis, Acute coronary syndrome, Infective endocarditis team, Surgery

## Abstract

**Introduction:**

Acute coronary syndrome (ACS) is an uncommon complication associated with high mortality in patients with endocarditis. It requires prompt and appropriate management to cure the patient.

**Cases presentation:**

We report the case of a 52-year-old patient, initially admitted for an acute non-ST-segment elevation coronary syndrome at very high ischemic risk, in whom coronary exploration was negative, and whose echocardiography showed a mobile image on the aortic valve, suggesting infective endocarditis. The patient benefited from an aortic valve replacement because of the size and the embolic complications he presented, with a favorable evolution.

**Discussion:**

Acute coronary syndrome during infective endocarditis is a rare complication with a high mortality rate. Several mechanisms are possible: the embolic mechanism, coronary extraluminal compression due to coronary mycotic aneurysm and obstruction of the coronary ostium by a large vegetation. The management remains multidisciplinary and personalized according to the phenotype of the patient, with the need to have the endocarditis team to be able to take the best therapeutic choice.

**Conclusion:**

Infective endocarditis must be evoked in any patient without usual cardiovascular risk factors who presents with an ACS that is accompanied by fever and elevated inflammatory markers, and a thorough clinical examination as well as the performance of additional tests.

## Highlights

• Acute coronary syndrome (ACS) is an uncommon complication associated with high mortality in patients with endocarditis.
• Our case presents a diagnostic challenge in the face of an initial misleading clinical presentation evoking ACS in a patient finally diagnosed with infective endocarditis.
• The management of endocarditis remains multidisciplinary, between cardiologist, infectiologist, radiologist, resuscitator and heart surgeons.

## 1 Introduction

Acute Coronary Syndrome (ACS) is an uncommon complication associated with high mortality in patients with endocarditis. It requires prompt and appropriate management to cure the patient. The management of a patient with infective endocarditis (IE) presenting with ACS requires an endocarditis team, including the specialties of interventional and non-interventional cardiology, cardiothoracic surgery, and infectious diseases. Our case presents a patient who presented with coronary syndrome in the setting of infective endocarditis of the aortic valve.

## 2 Cases presentation

**Fig. 1:**
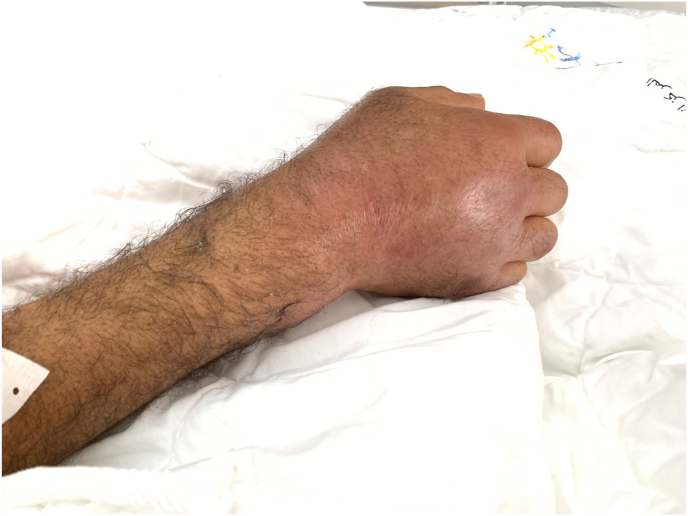
Presence of an inflammatory placard in front of the right wrist joint, with redness.

**Fig. 2:**
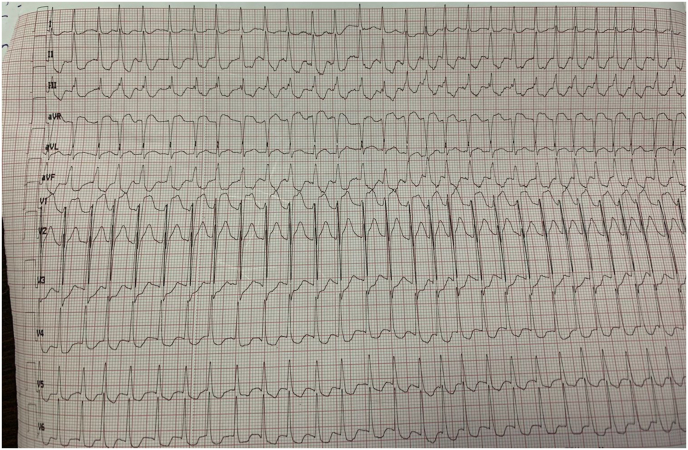
ECG showed an atrial fibrillation rhythm with a heart rate of 154 bpm, with a diffuse ST-segment undershift (more than 6 leads) and an ST-segment overshift in the anterior territory.

**Fig. 3:**
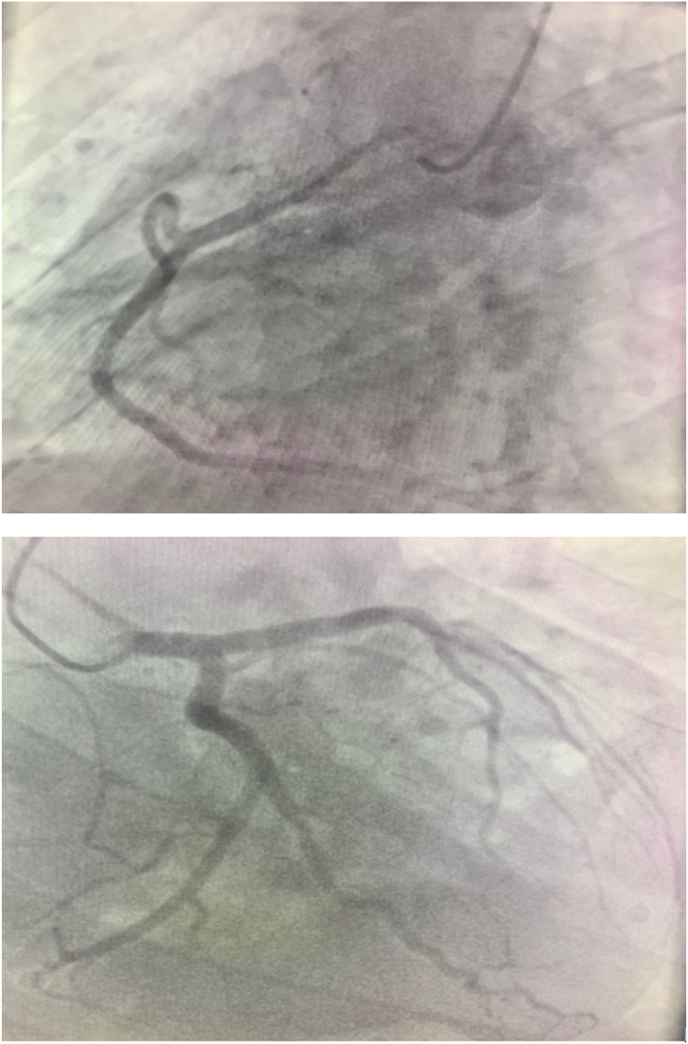
The coronary angiography found an angiographically normal coronary.

**Fig. 4:**
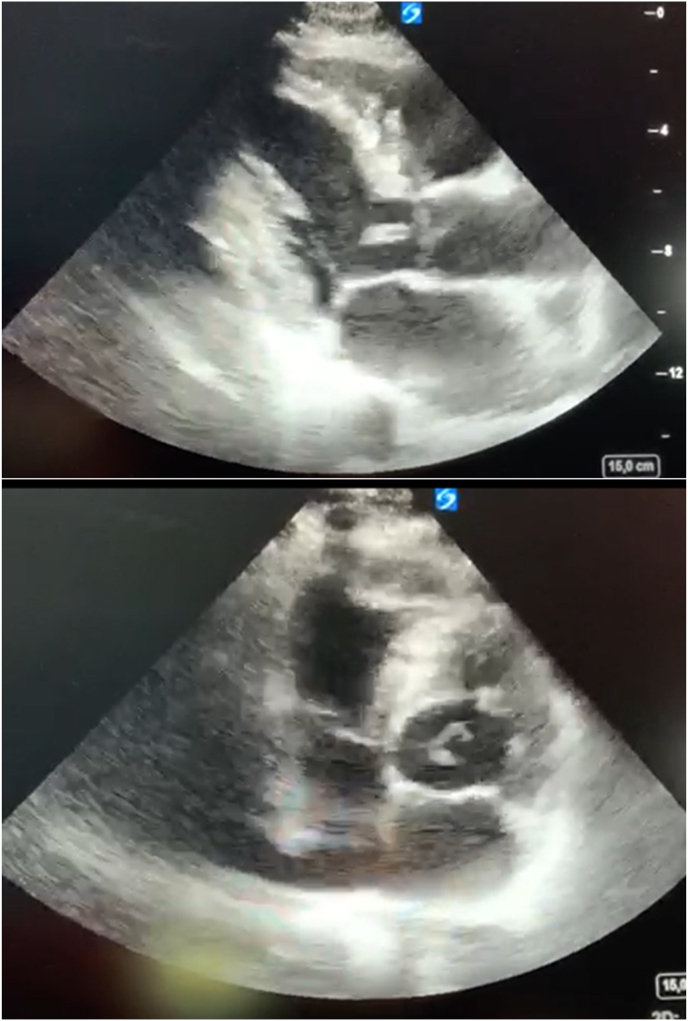
Echocardiography showed tri-commissural aortic valve with two vegetations appended respectively to the left coronary and non-coronary cusps measuring 3*34 mm and 4*41 mm in large diameter, with no significant leakage or stenosis No image of abscess or valvular destruction.

We report the case of a 52-year-old patient, with active smoking as cardiovascular risk factor, admitted to the emergency department for acute chest pain of infractoid appearance, established 5 hours before admission. The admission examination found a fast patient with a heart rate of 153bpm with normal blood pressure (123/76mmhg), respiratory and neurological stability, and a fever of 38.9°. The clinical examination did not reveal any signs of cardiac insufficiency, the cardiopulmonary ausculation was normal, but we noted the presence of an inflammatory placard in front of the right wrist joint, with redness and limitation of active and passive mobility that were painful (Fig. 1). The ECG showed an atrial fibrillation rhythm with a heart rate of 154 bpm, with a diffuse ST-segment undershift (more than 6 leads) and an ST-segment overshift in the anterior territory (Fig. 2). Given the clinical presentation and the electrical appearance, the diagnosis of an acute coronary syndrome without ST-segment elevation at very high ischemic risk was made, and the patient was sent directly to the catheterization room. The coronary angiography found an angiographically normal coronary network (Fig. 3), thus eliminating our initial diagnostic hypothesis. The biological workup found an elevated CRP of 456mg/l (Nvalue 6–12mg/l), a hyperleukocytosis of 22376 elements/mm3 (Nvalue 4000–1000/mm3) with PNN predominance, severe renal insufficiency with a creatinine of 24mg/l (Nvalue 6–12mg/l) and a clearance according to MDRD of 24ml/min. A *trans*-thoracic echocardiography (Fig. 4) was performed and found normal segmental and global right and left ventricular function with an EF estimated at 57% in SBP, with a tri-commissural aortic valve with two vegetations appended respectively to the left coronary and non-coronary cusps measuring 3*34 mm and 4*41 mm in large diameter, with no significant leakage or stenosis No image of abscess or valvular destruction. An infective endocarditis is strongly suspected, for that we realized 3 hemocultures at 1h of interval coming back negative, the rheumatoid factor is positive, the fractions of the complement C3 and C4 are normal, The realization of an injected Cerebro-thoraco-abdomino-pelvic scanner finds an aspect of splenic infarction with an articular effusion of the right glenohumeral joint associated with a thickening and enhancement of the synovial membrane making evoke in first a septic arthritis, Brain MRI found signalling abnormalities of the left basi-frontal subcortical white matter in favor of microfocuses of vascular accidents that may be related to septic microemboli. Given the presence of a vegatation on the aortic valve, fever, embolic syndrome and inflammatory phenomena, the diagnosis of infective endocarditis was made according to the modified Duke criteria (1 major and 3 minor criteria), the patient was put on antibiotics based on amoxicillin and Gentamycin (dose adapted to the renal function), and given the size of the vegatation and the embolic complications, the patient was referred to surgery.

On day 4 of his admission, the patient underwent an aortic valve replacement with a mechanical SJM valve and a vegectomy, with simple postoperative follow-up and rapid withdrawal of vasopressors. The culture of the vegetations came back inconclusive, then the patient was put on Vancomycin acenocoumarol 2mg/d, ceftriaxone 2g/d for 6 weeks with good clinical evolution and normalization of the biological balance at D20 of his admission.

The cytobacteriological examination of urine came back sterile, with no sign of intravenous drug abuse. The stomatological check-up showed a poor oral condition. The patient benefited from oral care. The patient was discharged home on day 26 of his admission with a negativation of the infectious balance sheet including procalcitonin at day 20.

## 3 Discussion

Infective endocarditis is a multi-systemic disease that is fatal if left untreated, and is defined as an infection of the endocardial surface including the heart valves, the mural endocardium or a septal defect.

Acute coronary syndrome during infective endocarditis is a rare complication with an incidence of 2.9% [1], and a mortality rate of 64%, it can reveal the infection and be the initial manifestation, recur a few days after the diagnosis of IE or at the end of treatment. Increased troponin T levels in endocarditis patients were a predictor of increased mortality and stroke [2].

This association can be explained by several mechanisms:

(a) Embolic mechanism: coronary emboli of septic origin usually occur within the first 2 weeks of infection.It is more frequent for fungal endocarditis than bacterial endocarditis because of the larger size of the vegetation. The risk of coronary embolism is increased when the vegetation is mobile, or it is larger than 10 mm, Staphylococcus aureus or streptococcus non viridans streptococcus infections and anterior embolism [3,4].This can be explained by the proximity between aortic vegetations and coronary ostia [4,5].
b) Coronary extraluminal compression due to coronary mycotic aneurysm or peri-annular aortic complication (abscesses and pseudoaneurysms), which are also described as a mechanism of myocardial ischemia during AR [6].
c) Obstruction of the coronary ostium by large vegetation [7], as in cases of fungal endocarditis with large vegetations.
d) Certain situations present during AR alter the perfusion/metabolic demand balance leading to onsite myocardial ischemia; anemia, fever, and activation of the coagulation system in septic patients and the presence of severe acute aortic valve regurgitation which in itself can cause myocardial ischemia by decreased coronary perfusion pressure and reduced coronary reserve.

The management of ACS during infective endocarditis remains controversial. Once the diagnosis of endocarditis is made, empirical antibiotic therapy should be considered while awaiting blood culture results. Although antithrombotic agents have been used successfully in the prevention and treatment of coronary embolism associated with IE, their value remains limited because bacteremia alters hemostasis which may increase the risk of intracerebral hemorrhage due to intracerebral mycotic aneurysms and cerebral infarcts (sequelae of IE) and therefore antithrombotic therapy should be used with caution [8].

The presence of AR contraindicates the use of fibrinolytic agents due to the risk of bleeding, which makes coronary angiography a safe procedure according to some investigators with a risk of fragmenting the vegetation if the catheter comes into contact with the surface of the aortic valve or at the time of contrast injection [9]. Guidelines recommend diagnostic coronary angiography for patients with active AR with elevated troponin levels or acute left ventricular dysfunction for STEMI, and do not recommend coronary angiography for non-ST-segment elevation myocardial infarction [10].

Emboli are usually treated by balloon embolectomy or surgery with simultaneous coronary artery bypass grafting and valve replacement taking into account the age of the patient, comorbidities and severity of the valve dysfunction. However, both techniques may increase the risk of dissemination of infection and the risk of bacterial myocarditis which may lead to wall rupture, coronary stenting with a risk of stent infection and coronary rupture. (This is probably due to the fact that small vegetative emboli that are fractured and crushed against the blood vessel wall during the procedure [11].

In practice, when a patient presents with ACS associated with endocarditis, if the patient presents with signs of periannular complication on TEE, without ST-segment elevation on ECG, the patient will be referred for surgery without performing coronary angiography, because the ischemia is probably due to compression of the coronary ostium. On the other hand, if no abscess or pseudo aneurysm of the aortic valve is detected or in the presence of an elevation of the ST segment on the ECG, a diagnostic coronary angiography is performed and an angioplasty with placement of a sent is performed if the artery is occluded, followed by an angiography in search of a mycotic aneurysm if the latter is present a surgical intervention is the rule [12].

The SCARE guildlines were used in the writing of this paper [13].

## 4 Conclusion

Infective endocarditis must be evoked in any patient without usual cardiovascular risk factors who presents with an ACS that is accompanied by elevated inflammatory markers, and a thorough clinical examination as well as the performance of additional examinations.

## Funding

This research did not receive any specific grant from funding agencies in the public, commercial, or not-for-profit sectors.

## Ethical approval

The ethical committee approval was not required give the article type (cases series).However, the written consent to publish the clinical data of the patients was given and is available to check by the handling editor if needed.

## Please state any sources of funding for your research

None.

## Author contribution

SOUMIA BOULOUZI: study concept or design, data collection, data analysis or interpretation, writing the paper. AMINE BOUCHLARHEM: data collection, data analysis or interpretation, writing the paper. SAIDA AMAQDOUF: data collection and data analysis. NOHA EL OUAFI: supervision and data validation. ZAKARIA BAZID: supervision and data validation.

## Please state any conflicts of interest

None.

## Consent

Written informed Consent was obtained from the patients for publication of this cases series and accompanying images. A copy of the written consent is available for review by the Editor-in-Chief of this journal on request.

## Registration of research studies

This is not an original research project involving human participants in an interventional or an observational study but a cases series. This registration is was not required.

## Guarantor

Soumia Boulouiz.

## Provenance and peer review

Not commissioned, externally peer reviewed.

## Declaration of competing interest

The authors declare no conflicts of interest.
